# Decodability, sensitivity, and criticality measured through single-neuron perturbations

**DOI:** 10.1038/s41467-026-69121-9

**Published:** 2026-02-13

**Authors:** Matthew Farrell, Taro Toyoizumi

**Affiliations:** 1 Laboratory for Neural Computation and Adaptation, RIKEN Center for Brain Science, Wako, Saitama Japan; 2 Department of Mathematical Informatics, Graduate School of Information Science and Technology, The University of Tokyo, Bunkyo-ku, Tokyo Japan

**Keywords:** Neural circuits, Network models, Dynamical systems

## Abstract

This comment highlights a new study by Ribeiro et al.^1^ which investigates how single-neuron spikes influence the surrounding cortical network in vivo. By comparing induced and background spikes through the lenses of decodability, sensitivity, and criticality, this work highlights how local perturbations interact with ongoing network dynamics to reveal multi-faceted signatures of critical neural computation.

## Neural decodability, sensitivity, and criticality

Neural network behavior can be decomposed along at least two dimensions: spatially (across the population) and temporally. The most local event in both senses is the activity of a particular neuron at a particular point in time. How such an atomic event influences the global state of the network is a question of fundamental interest across many fields, including neuroscience, physics, and machine learning. The activity of a given neuron is naturally shaped by ongoing network dynamics (cf. ref. ^2^); however, additional insight may be gained by experimentally driving neurons in controlled ways while observing the resulting impact on the surrounding network. This level of perturbative and observational control approaches that of model systems such as spin glasses and artificial neural networks, which may encourage a confluence of perspectives and techniques used for interpreting biological and artificial systems. We believe that the work highlighted in this comment^1^ is a compelling example of this confluence. Understanding the basics of network dynamical properties can lay a foundation for helping us understand how network activity impacts downstream circuits and, ultimately, behavior. Perturbations play an important role both in understanding basic dynamical properties of a neural network as well as their downstream computational impacts. “Atypical” perturbations from “typical” dynamics are often key for teasing out the behavioral role for a neural circuit; for instance, lesions of a brain area and the resulting impact on other neural systems and behavior have historically been powerful means to understand the roles of these brain areas. Perturbation at the level of single neurons is a technologically remarkable refinement of this basic principle, which is being used to explore the fine structure of network dynamics and the behaviorally relevant features of these dynamics. Theoretical work has laid important conceptual groundwork for linking local events to the global network responses they elicit. The featured work^1^ explores three related ideas: decodability, sensitivity of network dynamics to perturbations, and criticality^3–5^. Neural decodability generally refers to the ability to extract meaningful information from the neural population about what is going on in the real world, such as a sensory stimulus, a behavior, or, in this case, a direct perturbation of a neuron, based on spatial-temporal patterns of neuronal spiking^6,7^.

In^1^ spike decodability specifically tests the ability of a decoder to recover the identity of the neuron that emitted a spike by detecting the ongoing behavior of the surrounding network. Sensitivity measures how a small change (such as a spike caused by a perturbation) evolves through time, comparing the changed and unchanged network dynamics. Criticality places specific constraints on how activity propagates through a network and has been argued to achieve reliable information coding, propagation, and retention (a variety of formal definitions exist; below, we touch on a more concrete treatment). Experimentalists have increasingly drawn on these three theoretical frameworks to probe brain function. Early approaches to measuring sensitivity to input perturbations used patch-clamp stimulation of single neurons to quantify the sensitivity of network states to single-spike perturbations^8^. Other studies used multi-electrode recordings to observe population activity during sensory stimulation, such as whisker deflection in rodents^9^. In vitro experiments have enabled more fine-grained investigations of these ideas, albeit at the cost of natural ongoing dynamics^10^. Other studies have sought to characterize criticality with ongoing neural dynamics by measuring the size and duration distributions of neural “avalanches”^11,12^.

## Probing decodability, sensitivity, and criticality

The authors of^1^ extend this line of research by optogenetically stimulating individual neurons while recording surrounding network activity in vivo, enabling a more direct assessment of how individual spikes influence global network dynamics. The primary novelty lies in comparing signatures of decodability, sensitivity, and criticality between optogenetically induced spikes and spikes arising from ongoing “background” activity. This distinction is of interest to researchers across a broad spectrum, from applied fields such as brain–computer interfaces (BCIs) to theoretical studies of neural networks and criticality in physics-inspired systems. As such, we expect the results presented here to stimulate new perspectives across many domains. Below, we briefly summarize the main findings and consider some of their possible implications. The authors of^1^ find that the identity of an optogenetically driven neuron is, on average, much easier to decode than that of a neuron that spikes as part of ongoing dynamics. They also reproduce previously observed power-law statistics of spike avalanches, in which the sizes and durations of contiguous spiking events over the population follow power-law distributions. They then characterize network sensitivity by measuring how the mean size of a spike avalanche depends on the number of initial spikes of a selected neuron. The authors link these two measures—classical avalanche statistics and mean avalanche size as a function of number of initial spikes—conceptually by pointing to existing models of percolation and by simulation studies of a spiking neural network model. Interestingly, the mean avalanche scaling exponent differs between induced and background spikes: both exhibit power-law scaling, but with significantly different scaling exponents. The mechanisms underlying this phenomenon of distinct avalanche responses to induced versus background spiking are still mysterious, and our understanding would benefit from future exploration. Against this backdrop, the difference in decodability highlights the multi-faceted nature of criticality. As previously mentioned, the authors of^1^ reproduce experimental observations in a spiking neural network model, which is a helpful theoretical framework for thinking about these observations and why they occur. This elegant model does much to shed light on the experimental findings, while also leaving open interesting opportunities for further studies. For instance, the model here is tuned to reproduce the particular power-law scaling exponents as observed in experiments; we believe that it would be interesting to investigate mechanisms of self-tuning to criticality^3^ in the context of the observations made in this work. Another interesting point is that the model shows different mean avalanche scaling exponents in the high-baseline firing rate regime compared to the low-firing rate regime, unlike in the experimental observations (Supplementary Fig. 12 of ref. ^1^). Finally, due to efforts by the authors to match experimentally observed firing rates, excitatory external drive becomes vanishingly small when the network crosses into the supercritical regime, resulting in the network activity randomly dying out. An adjusted model may allow passing into the supercritical regime in a way that is more faithful to neural activity. We believe these aspects would be interesting to investigate in follow-up modeling studies.

## Advancing conceptual understanding of neural circuits

**Fig. 1:**
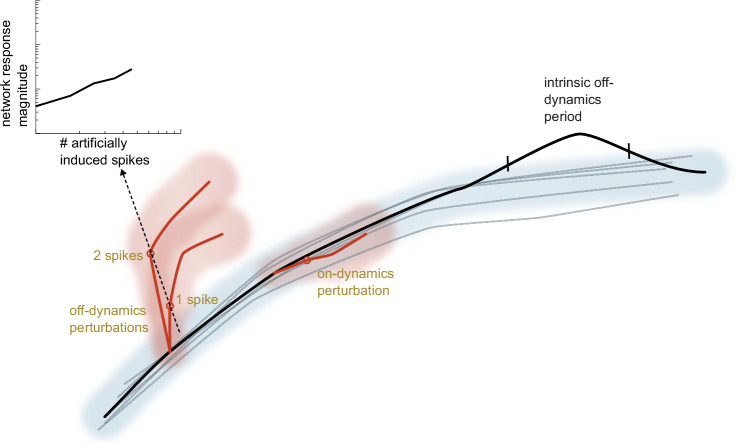
Illustration of neural trajectories during perturbation experiments. Depiction includes the following elements: an unperturbed intrinsic neural trajectory (black curve) alongside an ensemble of other non-perturbed trajectories (gray curves), mostly contained within a region of high probability (gray shaded region); externally induced spikes (red) that are both on-dynamics (within typical dynamics) and off-dynamics (away from typical dynamics), and which can be one or more spikes; deriving scaling laws for the average avalanche size in response to these induced spikes, as a function of number of induced spikes (offset panel); and an intrinsic off-dynamics period, which represents a time period in which the black neural trajectory naturally deviated from the region of high probability without external perturbation.

These findings may provide fodder for conceptual advances in how we think of neural network behavior. For instance, a popular framework for organizing neural activity is in terms of an “intrinsic manifold”, typically defined as a low-dimensional subspace that in some sense best contains the neural activity. When a neural population is driven within this intrinsic manifold, such as through BCI methods, animals appear to have a much easier time learning based on these perturbations^13^. Traditionally, the dimensions of the intrinsic subspace are estimated from the correlation structure of the neural data, which discards some temporal information. In reality, neural dynamics are characterized by specific trajectories with directionality on a manifold. Using the featured work^1^ as a starting point, it may be possible to define a notion of “on-dynamics” versus “off-dynamics” perturbations (Fig. 1). From this view, the induced spikes considered in this work would typically be off-dynamics, while background spikes would typically be on-dynamics; however, it may be possible to induce spikes in an on-dynamics fashion, and some background spikes may, by chance, be atypical enough to be considered off-dynamics. We believe that this would be an intriguing direction to look in. Mathematically speaking, decomposing a complex phenomenon in terms of local events and rules for how these events propagate is a powerful and important approach. However, how precisely to leverage perturbations to tease out information about neural circuits is an evolving theory. The work featured here^1^ takes an important conceptual step by comparing perturbations (induced spikes) to analogous non-perturbed events (background spikes). Follow-up studies could connect to other types of perturbations, such as on the basis of neuron type, or perturbations via ongoing spike train inputs^14–16^. Theory and experiment will continue to guide our exploration of what aspects of network dynamics are salient to downstream circuits and ultimately behavior^17^, and in turn our exploration of what kind of perturbations are meaningfully impactful.

## References

[1] Ribeiro, T. L. et al. Critical scaling of novelty in the cortex. *Nat. Commun.* (2026). 10.1038/s41467-025-68277-0 PMC12894744 41519964

[2] Luczak, A., Barthó, P. & Harris, K. D. Spontaneous events outline the realm of possible sensory responses in neocortical populations. *Neuron* **62**, 413–425 (2009). 19447096 10.1016/j.neuron.2009.03.014 PMC2696272

[3] Bertschinger, N. & Natschläger, T. Real-time computation at the edge of chaos in recurrent neural networks. *Neural Comput*. **16**, 1413–1436 (2004). 15165396 10.1162/089976604323057443

[4] Bertschinger, N., Natschläger, T. & Legenstein, R. At the edge of chaos: real-time computations and self-organized criticality in recurrent neural networks. In *Proc. Advances in Neural Information Processing Systems* Vol. 17 (MIT Press, 2004).

[5] Toyoizumi, T. & Abbott, L. F. Beyond the edge of chaos: amplification and temporal integration by recurrent networks in the chaotic regime. *Phys. Rev. E* **84**, 051908 (2011). 10.1103/PhysRevE.84.051908 PMC5558624 22181445

[6] Dayan, P. & Abbott, L. F. Chapter 3: Neural decoding. in *Theoretical Neuroscience, Computational and Mathematical Modeling of Neural Systems* (MIT Press, 2005).

[7] Zhu, Z. et al. Recent advances in spike-based neural coding for tactile perception. *Microsyst. Nanoeng.* **11**, 212 (2025). 41219192 10.1038/s41378-025-01074-3 PMC12606268

[8] London, M., Roth, A., Beeren, L., Häusser, M. & Latham, P. E. Sensitivity to perturbations in vivo implies high noise and suggests rate coding in cortex. *Nature* **466**, 123–127 (2010). 20596024 10.1038/nature09086 PMC2898896

[9] Gautam, S. H., Hoang, T. T., McClanahan, K., Grady, S. K. & Shew, W. L. Maximizing sensory dynamic range by tuning the cortical state to criticality. *PLOS Comput. Biol.* **11**, e1004576 (2015). 26623645 10.1371/journal.pcbi.1004576 PMC4666488

[10] Shew, W. L., Yang, H., Petermann, T., Roy, R. & Plenz, D. Neuronal avalanches imply maximum dynamic range in cortical networks at criticality. *J. Neurosci.* **29**, 15595–15600 (2009). 20007483 10.1523/JNEUROSCI.3864-09.2009 PMC3862241

[11] Gireesh, E. D. & Plenz, D. Neuronal avalanches organize as nested theta- and beta/gamma-oscillations during development of cortical layer 2/3. *Proc. Natl. Acad. Sci. USA* **105**, 7576–7581 (2008). 18499802 10.1073/pnas.0800537105 PMC2396689

[12] Kuśmierz, Ł., Ogawa, S. & Toyoizumi, T. Edge of chaos and avalanches in neural networks with heavy-tailed synaptic weight distribution. *Phys. Rev. Lett.* **125**, 028101 (2020). 32701351 10.1103/PhysRevLett.125.028101

[13] Sadtler, P. T. et al. Neural constraints on learning. *Nature* **512**, 423–426 (2014). 25164754 10.1038/nature13665 PMC4393644

[14] Legenstein, R. & Maass, W. Edge of chaos and prediction of computational performance for neural circuit models. *Neural Netw.* **20**, 323–334 (2007). 17517489 10.1016/j.neunet.2007.04.017

[15] Lajoie, G., Thivierge, J.-P. & Shea-Brown, E. Structured chaos shapes spike-response noise entropy in balanced neural networks. *Front. Comput. Neurosci.* **8**, 123 (2014). 25324772 10.3389/fncom.2014.00123 PMC4183092

[16] Lajoie, G., Lin, K. K., Thivierge, J.-P. & Shea-Brown, E. Encoding in balanced networks: revisiting spike patterns and chaos in stimulus-driven systems. *PLOS Comput. Biol.* **12**, e1005258 (2016). 27973557 10.1371/journal.pcbi.1005258 PMC5156368

[17] Marshel, J. H. et al. Cortical layer–specific critical dynamics triggering perception. *Science* **365**, eaaw5202 (2019). 31320556 10.1126/science.aaw5202 PMC6711485

